# Application of Red Cabbage Anthocyanins as pH-Sensitive Pigments in Smart Food Packaging and Sensors

**DOI:** 10.3390/polym14081629

**Published:** 2022-04-18

**Authors:** Reza Abedi-Firoozjah, Shima Yousefi, Mahshid Heydari, Faezeh Seyedfatehi, Shima Jafarzadeh, Reza Mohammadi, Milad Rouhi, Farhad Garavand

**Affiliations:** 1 Student Research Committee, Department of Food Science and Technology, School of Nutrition Sciences and Food Technology, Kermanshah University of Medical Sciences, Kermanshah 6715847141, Iran; r.abedi1878@gmail.com (R.A.-F.); mdheydari1371@gmail.com (M.H.); faeze_fatehi74@yahoo.com (F.S.); 2 Department of Agriculture and Food Science, Islamic Azad University, Science and Research Branch, Tehran 1477893855, Iran; shyousefi81@gmail.com; 3 School of Engineering, Edith Cowan University, Joondalup, WA 6027, Australia; s.jafarzadehkhosravi@ecu.edu.au; 4 Department of Food Science and Technology, School of Nutrition Sciences and Food Technology, Research Center for Environmental Determinants of Health (RCEDH), Health Institute, Kermanshah University of Medical Sciences, Kermanshah 6715847141, Iran; r.mohammadi@kums.ac.ir; 5 Department of Food Chemistry and Technology, Teagasc Moorepark Food Research Centre, P61 C996 Co. Cork, Ireland

**Keywords:** intelligent packaging, active packaging, *Brassica oleracea*, natural pigment, pH-responsive indicator

## Abstract

Anthocyanins are excellent antioxidant/antimicrobial agents as well as pH-sensitive indicators that provide new prospects to foster innovative smart packaging systems due to their ability to improve food shelf life and detect physicochemical and biological changes in packaged food. Compared with anthocyanins from other natural sources, red cabbage anthocyanins (RCAs) are of great interest in food packaging because they represent an acceptable color spectrum over a broad range of pH values. The current review addressed the recent advances in the application of RCAs in smart bio-based food packaging systems and sensors. This review was prepared based on the scientific reports found on Web of Science, Scopus, and Google Scholar from February 2000 to February 2022. The studies showed that the incorporation of RCAs in different biopolymeric films could affect their physical, mechanical, thermal, and structural properties. Moreover, the use of RCAs as colorimetric pH-responsive agents can reliably monitor the qualitative properties of the packaged food products in a real-time assessment. Therefore, the development of smart biodegradable films using RCAs is a promising approach to the prospect of food packaging.

## 1. Introduction

Food packaging is considered an important parameter in preserving food quality and safety mainly by controlling the oxygen transportation mechanism as well as the inhibition of microbial entry, providing a suitable covering to support the food integrity and extend the product shelf life [1,2]. Petroleum-based packaging materials are well-known cost-effective ingredients protecting food items against physicochemical damages and harsh microbial and environmental conditions [3,4]. However, the widespread consumption of petroleum-derived plastics has become a serious global concern due to their environmental problems in production and disposal. In addition, they cannot satisfy the consumer’s demands for safe and quality food. Hence, a growing interest has been created in the application of naturally biodegradable and edible packaging materials (e.g., proteins, polysaccharides, and lipids) as raw materials from natural and biological resources [1,5].

To reduce the environmental and health problems and increase the efficiency of packaging, modern-day bio-based packaging is nowadays widely developed in the world [6,7]. In this regard, many researchers have tried to find innovative food edible packaging based on various types of natural biopolymers to reduce the health risks associated with the petroleum-based polymer residues that migrate to the packaged food [8,9,10]. Over the past two decades, innovation in food packaging is principally attributed to the development of smart packaging techniques, which can be placed in two principal categories: active and intelligent packaging. Active packaging systems are fabricated by loading functional bioactive components such as natural plant extracts, essential oils, antimicrobials, etc. to achieve a prolonged shelf life in food products [9,10,11]. The real-time monitoring of the food quality and storage situations from food plants to the end-user is also defined as intelligent food packaging [10,12]. Hence, smart food packaging systems are generated based on the effective correlation among food products, packaging materials, internal and external environmental factors, and consumers. It is developed based on quality sensors, indicators, and traceability methods to inform consumers of some beneficial information about the food quality and safety changes throughout the food supply chain. 

In addition to their beneficial health properties, most natural colors have revealed temperature, freshness, gas, or pH-responsive properties, which could be used as colorimetric sensors/indicators. The growing demand for consuming natural/organic products can consider these natural pigments as a healthier and safer replacement for synthetic colorants [13]. Therefore, the development of biodegradable smart packaging systems equipped with colorimetric indicators based on pH-sensitive natural colors has recently received increasing attention as a promising substitute in food packaging systems because of their low cost, availability, abundance, and reliable halochromic (pH-sensitive) capacity [14,15,16,17].

The pH indicator during the food supply chain is of importance because it exhibits color change as a result of changes in pH arising from chemical reactions or microbial growth [12,18]. The spoilage of food products is directly associated with the pH alterations of the product. As a result, employing smart packaging films containing natural colorants is proposed as a suitable alternative for consumers to assess food quality and safety in real time [19,20].

Among the natural colorants, anthocyanins have been broadly utilized in smart food packaging composites and colorimetric sensors as halochromic colorants [21,22]. They are water-soluble flavonoid pigments that reflect light in the red–blue range in the visible spectrum [23]. Anthocyanins could represent the chemical and sometimes microbial changes within food products as per their pH-sensitive nature. Furthermore, anthocyanins, possess excellent antimicrobial and antioxidant activities, making them excellent candidates to extend the shelf life of food products [24,25]. Structurally, anthocyanins are composed of the glycosylated structure of anthocyanidins. Typically, the most widely used types of anthocyanidins found in the environment include delphinidin, peonidin, cyanidin, malvidin, pelargonidin, and petunidin [26,27]. Recently, anthocyanins extracted from different plant sources have been incorporated into polymer-based films to design and develop active and/or intelligent packaging films due to their radical scavenging, antimicrobial, and pH-responsive color-changing properties [16,28]. 

Different types of polysaccharides (such as chitosan, starch, cellulose, pectin, and some natural gums), proteins (such as zein, soy protein isolate, and gelatin), and biocompatible synthetic polymers (such as PVA and PLA) have been selected as the single or composite matrix of the anthocyanins-rich packaging films. To overcome the inferior or limited functional properties of single biopolymeric smart films, several approaches including the development of composite films, incorporation of nanomaterials, and/or use of crosslinkers have been introduced [29,30,31]. In addition, different procedures, such as casting, extrusion, and electrospinning methods have been used for the preparation of anthocyanins-rich films [16,28,31,32,33]. 

On the other hand, the anthocyanins incorporated into the films have been isolated from various plant sources such as red cabbage, red radish, red grapes, cherries, berries, black rice bran, black/purple eggplants, black plum, purple sweet potato, and roselle [28,34]. It has been indicated that anthocyanins from different plant sources have distinct pH-sensitivity properties [16,28], probably due to the differences in their content, composition, extraction method, and polymeric habitation. Several studies also reported that the mechanical, physical, thermal, and structural properties of the films can be negatively/positively affected by anthocyanins [28,35,36]. However, no studies have reviewed the functional properties of different types of smart films containing anthocyanins from a specific source such as red cabbage.

Red cabbage (*Brassica oleracea* L.) is introduced as one of the typical sources of natural anthocyanins, possessing good nutritional value and positive effects on human health because it is rich in micronutrients and some phytochemicals including oligosaccharides, minerals, vitamins, and some bioactive compounds such as high levels of anthocyanins, flavonols, and glucosinolates [37,38]. Red cabbage extract (RCE) is a common and rich source of anthocyanins, which are closely related to the pH of their environment, ranging from red color at pH 1–2, pink at pH 3, violet at pH 4–6, blue at pH 7–8, green at pH 9–11, and yellow at pH 12. The RCE is naturally abundant and its preparation cost is lower than other plant sources. Thus, these properties make red cabbage anthocyanins (RCAs) suitable for application in pH-responsive indicator films [39]. Compared with anthocyanins from other natural sources, red cabbage anthocyanins have received much attention from researchers due to their low cost, availability, abundance, and reliable halochromic capacity [40,41]. The antioxidant/antimicrobial properties as well as pH-sensitivity of RCAs are critical attributes to developing active and intelligent packaging systems [39]. 

The core objective of the current review was to investigate the latest research findings on the pH-diagnosing smart systems based on red cabbage anthocyanins and the effects of their incorporation on the physical, mechanical, thermal, and structural properties of packaging films, as well as their potential applications in food packaging systems and biosensors.

## 2. Halochromic (pH-Sensitive) Properties of Red Cabbage Anthocyanins

Generally, changes in the safety and quality of food products can occur during production, storage, distribution, shipment, and consumption. Consumers commonly detect and evaluate the freshness and quality of packaged foods using the shelf life date printed on the package. However, the shelf life date alone cannot be enough to evaluate the freshness and quality of some food products such as fresh fruits and vegetables [42]. Color, as a natural indicator of food quality, is regarded as one of the key factors to identify and monitor the physicochemical alterations of food products [13,22]. In this regard, the use of intelligent color sensors and labels is an innovative and smart system for detecting, tracking, protecting, and assuring production of safe and high-quality products. The utilization of natural colorants (mainly plant-based ones) from various sources has been suggested as proper alternatives to unsafe synthetic (chemical) dyes. In recent years, the interaction (sensitivity) of natural colorants (especially anthocyanins) toward environmental variations, triggered a huge interest in their application as suitable tools to produce pH, gas, or temperature-responsive smart packaging systems [32,43]. 

**Figure 1:**
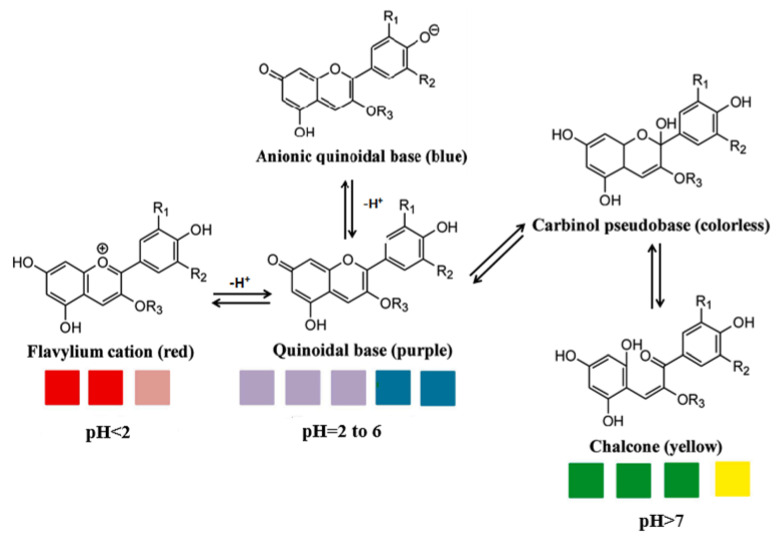
Color changes of red cabbage anthocyanin-rich extract at different pH values.

Anthocyanins, as one of the water-soluble phenolic compounds, are able to generate a wide range of colors (e.g., blue, purple, orange, and red) that are widely isolated from flowers, cereals, fruits, and vegetables [44]. In addition, based on the pH values of the solution, anthocyanins can be found in different colors and chemical forms that can monitor food quality parameters, and eventually, keep track of food products over the shelf life period [18,45]. The reversible color attributes of anthocyanins-rich solutions are associated with the source, composition, and configuration of anthocyanins. Accordingly, four different chemical forms of these compounds with various colors are in different pH values of the solution (Figure 1) [14,46].

Based on Figure 1, the color variation of the RCA-rich extract is observed by a stepwise increase in pH from the acidic to the alkaline region. Due to the hyperchromic and bathochromic properties, the color of RCA-rich extract is varied from red to green/yellow at pH values of 2 to 12 [34,47]. At the lowest pH values (pH < 2), red dye caused by the flavylium cation is the predominant color. With a slight increase in pH (pH = 2–4), the color changes toward a purple/blue quinoidal base. Then, the colorless carbinol pseudo-base will be the dominant color by increasing pH from acidic to slightly acidic/near-neutral conditions. Further increase in pH value (pH > 7) causes a gradual decrease in anthocyanins stability and generates a green–yellow color as a result of chalcone formation. Thus, the pH-responsive activity of anthocyanins could be useful to foster intelligent packaging systems [41,47,48,49]. 

## 3. Effects of Red Cabbage Anthocyanins on Properties of Smart Bio-Based Films

### 3.1. Physical Properties

#### 3.1.1. Thickness

The thickness of packaging films is an important factor due to its impact on mechanical, light transmittance, and gas barrier properties of the fabricated composites. This factor is highly influenced by the film composition, dispersibility, and flow properties. Several studies confirmed that the low contents of anthocyanins cause no significant difference in film thickness because they can uniformly distribute in the film matrix [17,39,50,51,52]. Chen et al. [39] indicated that the film thickness did not significantly change by incorporation of various contents of RCAs, probably due to the low RCAs concentration, and proper compatibility of RCAs within the chitosan/oxidized chitin nanocrystals (CS/OCN) composite matrix. Similarly, Park et al. [53] reported no significant difference in the thickness of edible chitosan-based films, which could be due to the low amount and low dry matter content of the extract in film formulations. 

However, loading high amounts of anthocyanins could disrupt the integrity of the film matrix and influence the film thickness. In this regard, Prietto et al. [54] noted that the thickness of pH-sensitive corn starch films increased by the addition of anthocyanins content. In agreement with these results, the addition of RCE caused a linear increase in the thickness of colorimetric films based on polyvinyl alcohol/sodium carboxymethyl cellulose (PVA/CMC·Na) [55] and cellulose acetate [47]. In contrast, do Nascimento Alves et al. [56] stated that various RCE-loaded biodegradable films including polyvinyl chloride, gelatin, and green banana starch fabricated slender film layers compared with the control films without RCE. In accordance with these studies, it can be inferred that the incorporation of RCAs has different effects on film thickness in smart packaging films, probably depending on the composite matrix and the RCA characteristics (composition and amounts).

#### 3.1.2. Moisture Absorbency and Swelling Index

Moisture absorbency and swelling index (SI) are important factors for developing pH-sensitive smart packaging systems due to their influences on color response efficiency within the film matrix [57]. This is more critical in water-sensitive hydrophilic composite films since the moisture content and water activity could significantly alter their structure and functionality [58]. The higher SI value causes a rapid color release which is not a desirable reaction in colorimetric composite films [19,55].

do Nascimento Alves et al. [56] stated that the loading of RCE reduced the water absorption and SI in gelatin, alginate, and banana starch biopolymers. In contrast, Pourjavaher et al. [57] showed that the moisture absorption of bacterial cellulose (BC) nanofibers with various concentrations of RCA extract was higher than pure BC nanofibers because RCAs caused disturbed compactness of the polymer network in addition to the increased free volumes and cavities within the polymer matrix. Thus, the water vapor molecules absorbed into these voids led to increasing moisture absorption of nanofibers networks. Similarly, Kuswandi et al. [19] reported that the SI of the BC membrane with immobilized RCAs was significantly higher than the BC membrane. Maftoonazad and Ramaswamy [58] also confirmed that the moisture adsorption of PVA nanofiber increased by RCE incorporation. They suggested that the polyphenolic compounds of RCE probably caused the reduction in intermolecular interactions and cohesiveness in the PVA network, leading to an increase in moisture absorption of the PVA/RCE nanofiber network. Hence, RCA addition could increase the hydrophilic properties of biopolymer films. However, because of insufficient evidence, it is difficult to determine a definite effect of anthocyanins on moisture absorbency.

#### 3.1.3. Water Solubility

Water solubility reflects the water sensitivity of films. Smart packaging films with remarkable water resistance are preferred to preserve food items with intermediate or high moisture content. Intelligent films with low water resistance can dissolve quickly which causes a significant loss and release in colorimetric agents [59]. Wu et al. [52] reported that the water solubility of konjac glucomannan films with oxidized chitin nanocrystals (KGM/O-ChNCs) was significantly increased as a result of RCAs addition. This effect is correlated to the extremely hydrophilic nature of anthocyanins. Similarly, Prietto et al. [54] reported that the addition of RCAs in pH-sensitive corn starch films increased their water solubility due to the increased hydrophilic spots within the biopolymer matrix. They also reported that the acylation and glycosylation of anthocyanins can reduce and increase the water solubility of the films, respectively. Likewise, Kuswandi et al. [19] found that the addition of RCAs into the BC membrane significantly increased solubility in water. Thus, the interaction of RCAs with the film matrix often enhanced the water solubility of films due to their hydrophilic nature.

#### 3.1.4. Oxygen and Water Vapor Permeability

Oxygen permeability (OP) and water vapor permeability (WVP) are two determining factors to track the permeability attributes of food packaging systems as important criteria in food quality and safety [39]. They are of great importance in extending the shelf life of packaged food by maintaining a suitable equilibrium of moisture and oxygen contents and controlling physical or chemical deterioration. Prevention or reduction in moisture and oxygen transfer between the food and the surrounding environment is a primary function of food packaging and low WVP and OP are generally required for food packaging [50].

Liang et al. [60] reported that the WVP values of the composite films increased due to the rupture of the compact network between the composite film ingredients, namely, CMC·Na and *Artemisia sphaerocephala* Krasch. gum (ASKG), as well as the hydrophilic attributes of RCA. Their results also showed a significant drop in the OP of films as a result of RCA addition as the polarity of RCA blocks the oxygen penetration into the packaging film. Moreover, Wu et al. [52] reported an increased WVP in KGM/O-ChNCs composite films when loaded with RCA, which could be correlated with the plasticizing effects of RCA within the polymer chain. On the contrary, Chen et al. [39] presented that the addition of RCAs into CS/OCN composite films considerably decreased the WVP. They claimed that these variations could be attributed to the formation of hydrogen bonds between the composite film and RCAs, as well as the influence of the aromatic rings in the RCA structure which impacts the construction of a denser microstructure network, decreasing the binding affinity of the polymer chain toward the water molecules. They also reported that the incorporation of RCAs into composite film remarkably declined OP values. do Nascimento Alves et al. [56] also indicated that the WVP of the alginate/starch/gelatin film was significantly reduced with the addition of RCE, probably because the extract was not capable of chemically bonding with the polymers, specifically gelatin, which prevented the loss of cohesion of the film and consequently decreased the vapor diffusion of water. However, Silva-Pereira et al. [45] observed that the addition of RCE did not significantly change the WVP of the chitosan/corn starch blend films. 

The film permeability depends on many factors, such as the integrity and mobility of the polymeric chain, the ratio between crystalline and amorphous zones, the hydrophilic/hydrophobic ratio, etc. [61]. The presence of RCA in the biopolymeric film matrix could affect these factors and modify the WVP and/or OP properties.

### 3.2. Mechanical Attributes

The integrity and sustainability of food products could be guaranteed by composite films possessing suitable mechanical strength. Tensile strength (TS), and elongation at break (EAB) are two important mechanical criteria to monitor the strength and flexibility of packaging films, respectively. TS is defined as the maximum tolerance of composite films against the applied stress while being pulled or stretched before breaking occurs. Moreover, EAB is defined as the maximum capability of composite films to maintain the alterations in the length and shape of the films deprived of any crack formation [41,62].

Generally, the mechanical properties (TS and EAB) of RCAs-rich films can be affected by various factors. In this regard, Liang et al. [41] reported that the TS of the RCA-blended ASKG/CMC·Na composite films decreased by loading RCA, while their EAB showed an increasing pattern. It might be due to the formation of some interactions between RCA and composite matrix, and plasticizing effects of RCA, both varying the state of hydrogen bonds within the polymer chains, resulting in enhanced molecular mobility and hence a damaged integrity network [41]. Similarly, Park et al. [53] also indicated that the addition of RCA decreased the TS value and remarkably increased the EAB value of edible chitosan films. Pourjavaher et al. [57] reported that the mechanical properties of anthocyanins-rich BC nanofibers can be influenced by anthocyanins concentration. They observed a concentration-dependent decrease in the TS and an increase in EAB, which was in agreement with the study of Chen et al. [39] on chitosan/oxidized–chitin nanocrystals (CS/OCN) composite films and the study of Freitas et al. [47] on cellulose acetate-based films. On the other hand, the phenolic compounds of RCAs in the BC nanofibers matrix probably act as the plasticizer agent and reduce the interactions among the BC membrane macromolecules. Kuswandi et al. [19] observed similar effects of the RCA phenolic compounds on the mechanical properties of BC membrane.

However, in a study by Wu et al. [52], the incorporation of RCA in the KGM/O-ChNCs matrix led to decreases in both TS and EAB values because of the weakening of the compact structure of konjac glucomannan-oxidized chitin nanocrystals film. In contrast, Chu et al. [63] found that with the addition of up to 2 wt% red cabbage pigment to cationic guar gum/hydroxyethyl cellulose (CGG–HEC) composite films, both TS and EAB increased due to a good hydrogen bonding between RCA molecules and the composite matrix and then decreased. Thereafter, further addition of RCA (>2%) resulted in a decrease in both TS and EAB, due to more electrostatic repulsion of O+ in RCA and N+ in CGG.

In addition, the mechanical attributes of the RCAs-loaded composite films could be greatly affected by the anthocyanins source and the extraction procedure [54]. For instance, the gelatin-based smart packaging films containing the aqueous extract of anthocyanins exhibited a higher TS, while a lower EAB was observed when compared with the gelatin films containing alcoholic extract of anthocyanins [64]. Comparable findings were also noted for various packaging films containing anthocyanins extracted by various solvents [58,65].

Overall, the mechanical strength of RCAs-loaded packaging films could be influenced by various factors including the interaction between RCAs with the polymer functional groups, co-film-forming agents (e.g., plasticizers, nanoparticles, crosslinkers, etc.), and the water molecules. Electrostatic repulsions and hydrogens are key interactions associated with the bonding of RCAs and film components. The type of polymer and RCAs concentration could also impact the mechanical attributes of RCAs-loaded composite films [39]. 

### 3.3. Thermal Characteristics

Mapping the thermal degradation profile of composite films is introduced as an efficient tool to understand their thermal stability. For this purpose, thermogravimetric analysis (TGA) and differential scanning calorimetry (DSC) are usually employed to monitor the thermal stability of composite films. Some studies have shown that the incorporation of RCAs decreases the thermal stability of the films because the RCAs weaken the intermolecular interactions among film components, which facilitates its decomposition at lower temperatures. For instance, Silva-Pereira et al. [45] found that the addition of RCE into chitosan/starch film caused lower thermal stability compared with the control film. Similar findings were reported by Prietto et al. [54] in the starch films incorporated with RCE.

In contrast, some studies have informed that the thermal stability of composite films could be improved by loading RCAs-rich extracts due to the generation of robust intermolecular connections between RCAs and polymer chains [39]. Freitas et al. [51] reported that the thermal properties of hydroxypropyl methylcellulose (HPMC) packaging films were influenced by loading RCA, as well as the pH variations of the film-forming solution. Based on the results, the incorporation of RCA increased the film’s thermal stability and consequently exhibited higher maximum values of thermal degradation temperatures, which can be related to the formation of hydrogen bonds between anthocyanins and polymer chains, leading to a decrease in the accessibility of hydroxyl groups in HPMC that interact with water molecules. Chu et al. [63] also observed the maximum decomposition temperature of anthocyanins-rich composite cationic guar gum–hydroxyethyl cellulose films was significantly higher than other reference films. In line with these findings, Eskandarabadi et al. [66] reported an increase in the thermal stability of biodegradable ethylene-vinyl acetate nanocomposite films added with anthocyanin. However, Liang et al. [41] indicated that the incorporation of RCA didn’t change the thermal stability of the ASKG packaging films. As a result, the thermal properties of anthocyanins-loaded packaging films may be also affected by various factors such as polymer type, the interaction among film components, as well as the source and content of the incorporated anthocyanins.

### 3.4. Structural Properties

The structural properties of biopolymer films containing RCAs were commonly determined by FTIR analysis, which investigates the inter- and intra-molecular interactions between the composite film components. 

The characteristic FTIR bands of RCAs were introduced in some studies [51,55,58,60,67]. Freitas et al. [51] reported a broad strong absorption band at the wavenumber of 3360 cm^−1^, demonstrating the presence of O–H stretching vibration as a result of the formation of hydrogen bonds in RCE [57]. The peaks at about 2980 cm^−1^ and 1645–1735 cm^−1^ are assigned to C–H stretching vibration present in aromatic rings, and the stretching vibration of C=O flavonoids, respectively, confirming the presence of aromatic compounds in RCE. A peak at the range of 1620–1640 cm^−1^ is probably generated due to the formation of stretching vibrations of aromatic rings of C=C bands. The band at the range of 1410–1415 cm^−1^ belongs to the C–O groups which displays the angular deformation of the phenols [55]. However, Freitas et al. [51] found these groups in tiny absorption bands in the range of 1300–1380 cm^−1^. The peaks at about 1090 cm^−1^ and 1050 cm^−1^ are related to C–O–C stretching vibration and C–O–C–O–C alkyl aryl ether (or anhydroglucose ring of O–C) stretching vibration, respectively. The peaks exist in the cyanidin-3,5-O-diglucoside structure, known as the main cyaniding in red cabbage. The bands from 990 cm^−1^ to 1000 cm^−1^ are also assigned to C=O stretching vibrations. 

Though Hamzah et al. [67] reported no significant variations in the physical and chemical attributes of sage starch films containing RCA, most authors observed changes in biopolymer structures due to the addition of RCA to films through the formation of hydrogen bonds with the film matrix. Freitas et al. [51] observed some wavenumber shifts to slighter absorption positions in the O–H stretch region in HPMC film containing RCE, indicating the extract incorporation into the polymer matrix, and probably interaction by hydrogen bonds with functional groups present in the HPMC and the glycerol. Furthermore, Maftoonazad and Ramaswamy, Liu et al., and Liang et al. [55,58,60] confirmed the immobilization of RCA in biopolymer composite films by electrostatic interactions. 

Maftoonazad and Ramaswamy [58] observed several FTIR indications for successful immobilization of RCE in PVA biopolymers through the construction of intermolecular hydrogen connections between RCE and PVA chains, causing a remarkable decrease in the intensity of O–H peaks at 3000–3600 cm^−1^ and C=O stretching vibrations at 1735 cm^−1^, formation of an absorption band at 1669 cm^−1^ assigned to the C=O stretching vibrations, and nearly fading of the bands at 1247 cm^−1^ in the FTIR spectra of PVA-RCE composite film when compared with the pure RCE.

The FTIR spectrum of PVA/CMC·Na films containing RCAs in the study of Liu et al. [55] showed that the peak at 1091 cm^−1^ was gradually expanded by increasing the RCAs level within the composite film most probably as a result of concurrence of C–OC bonds in CMC·Na and C–OC bonds in RCAs. The peaks at 1598 cm^−1^ (COO—asymmetric stretch) and 1419 cm^−1^ (COO—symmetric stretch) asymmetrically expanded and displaced as per the formation of electrostatic interactions within RCAs, and also the formation of intermolecular hydrogen bonds between PVA and RCAs. Similar results were reported by Liang et al. [60], who found the breaking of the hydrogen bonds between ASKG and CMC·Na with the incorporation of RCA to ASKG/CMC·Na blended films because of the increase in bands at 2924 and 2879 cm^−1^. The bands at 1642 cm^−1^ (C=C—stretching vibration) and 1594 cm^−1^ (COO—asymmetric stretch) declined and expanded due to the CMC·Na-RCAs electrostatic interactions as well as the formation of RCAs-mediated hydrogen connections between CMC·Na and ASKG polymers. 

These researches showed that the RCAs were physically integrated into the biopolymeric matrix by hydrogen bonds, which are weaker than the covalent chemical bonds and have little or no effect on the halochromic properties of RCA.

## 4. Applications in Food Packaging and Sensors

The pH-sensitivity and color-changing properties of anthocyanins-rich films have been successfully employed to develop halochromic packaging films and monitor the freshness of food products in real time. For this reason, much effort has been made to integrate indicators/sensors based on the red cabbage anthocyanins into packaging materials. Recently, numerous kinds of biodegradable biopolymers (alone or in combination with different polymers) have been applied for the development of color indicator films incorporated with RCA (Table 1).

### 4.1. Intelligent Characteristics in Natural Biopolymeric Films

Natural biopolymers, especially polysaccharide and protein-based ones, have been widely used to produce the halochromic active and intelligent packaging films due to their biocompatibility/biodegradability, nontoxicity, stability, easy availability, and good film-forming ability. Kuswandi et al. [19] reported good performance of an edible pH sensor based on the immobilized RCA into BC membrane as it was employed to track the pH alterations of some beverages. Additionally, the pH sensor can be used for observing the milk freshness, as it can easily differentiate the deteriorated milk from fresh milk by using color detection sensors that display the pinkish-gray to the bluish-gray color range. In this regard, do Nascimento Alves et al. [56] fabricated biodegradable films using green banana starch, gelatin, and alginate incorporated with RCA for monitoring the quality of sheep meat freshness. They observed changes in color parameters due to the increase in the pH of the meat. The pH of sheep meat was increased due to the volatile alkaline compounds formed in the samples during the storage period and thereby all films indicated changes in color parameters.

**Figure 2:**
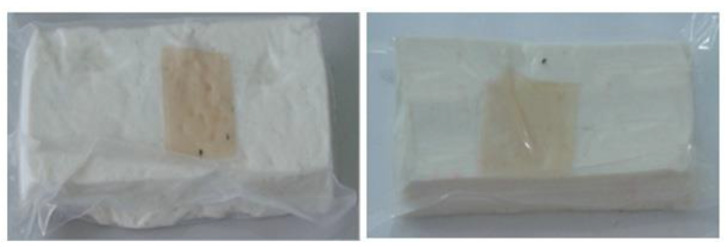
Color change in a pH-sensitive composite film produced from anthocyanins-loaded PVA–chitosan–gelatin for monitoring the spoilage of ricotta cheese during refrigerated storage: (**left**) 1 day (**right**) 7 days [73].

Chitosan is the most common natural biopolymer that has been used in the fabrication of smart films with RCA. Silva-Pereira et al. [45] monitored fish deterioration using chitosan/corn starch blended film with RCA. They observed color change in the film depending on the different conditions of the fish sample during storage at room temperature. Based on the result, no color change was observed in the indicator film after 12 h. After 16 h of storage, the color began to shift to blue, which showed initial spoilage and pH increase. After 72 h of storage, the color completely turned to yellow, indicating complete fish spoilage [45]. Bento et al. [73] developed and evaluated a pH-sensitive packaging film using chitosan, gelatin, PVA, and RCE to monitor ricotta cheese spoilage. Despite the high sensitivity of film to pH changes, the results demonstrated that the initial light brown color of the indicator film was not significantly changed after seven days of refrigerated storage, which may be correlated with keeping pH at 4.48 as well as viable numbers of mesophilic microorganisms compared with initial time (Figure 2). Recently, antimicrobial activity and indicator properties of edible chitosan-based films prepared with RCE (as spoilage indicator) and clove bud oil (CBO; as antimicrobial agents) were investigated by Park et al. [53]. They reported that the pH of fish peptone agar including *Pseudomonas fluorescens* enhanced from near 6 to 9, and the initial purple color of the films changed to deep blue during the growth of fish-spoiling bacteria. They claimed that the edible films containing CBO and red cabbage have a high potential for use in fish preservation. Vo et al. [65] tested the freshness of pork belly using an intelligent film prepared with chitosan/PVA/RCA for 24 h at room temperature. They observed that the initial sea green color of the film changed to pink color at 12 h, representing an acidic condition near pH 5–6 on the pork slices surface. After 24 h, the film wrapping the meat turned pale pink, displaying it in the slightly alkaline range. The color change appeared due to the meat spoilage during the growth of microorganisms and the deterioration of samples caused by biochemical reactions.

In recent years, gums, as polysaccharide compounds produced from plants, seeds, and microbial resources, have been used for the development of color indicator films [74,75]. Liang et al. [41] prepared an intelligent pH indicator film using the ASKG, carboxymethyl cellulose sodium, and RCA. They reported that the color of anthocyanin changed from pink to green when the pH changed from 3 to 10. Furthermore, films containing more than 5% RCA were better suited for practical applications due to the visible color changes. In another study, a cationic guar gum with hydroxyethyl cellulose, and red cabbage pigment was developed by Chu et al. [63] for the design and validation of antibacterial and pH-responsive film in detecting the deterioration of pork and soybean milk. Generally, the volatile alkaline substances generated via pork deterioration or microorganisms activity in soybean milk are regarded as the causes of pH alteration. They observed that the color of the indicator film with 10% hydroxyethyl cellulose and 3% red cabbage pigment was significantly changed over time [50].

### 4.2. Intelligent Characteristics in Nano-Biocomposite Films

**Figure 3:**
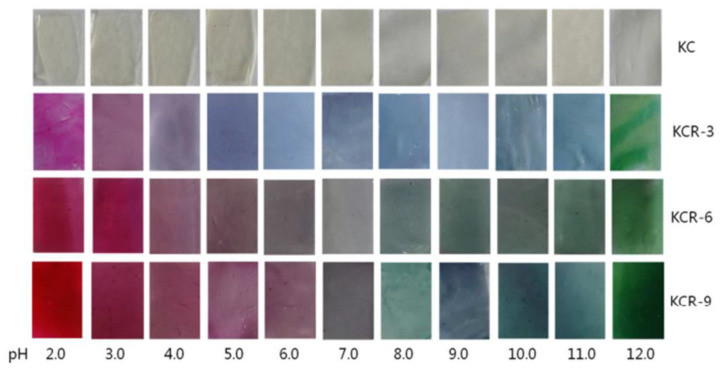
Color variation of composite films as exposed to different pH values (2–12) for 10 min. KC: konjac glucomannan films with oxidized chitin nanocrystals without RCA, KCR-3: film with 3% RCA, KCR-6: film with 6% RCA, and KCR-9: film with 9% RCA. Reprinted with permission from Ref. [52]. Copyright 2019 Elsevier Ltd.

In addition, some studies considered the nanocomposite matrix as the residence of RCA. Halochromic CS/OCN composite films and RCA were applied for monitoring the hairtail and shrimp freshness by Chen et al. [39]. The films exhibited visible color differences when the pH varied from 3 to 13. In addition, the film color was developed by increasing the anthocyanins level (0–1.2%, *w*/*v*), so that the color of the film with 1.2% RCA was distinctly different compared with the control film (without RCA) during 48 h of hairtail and shrimp storage at 25°C. They concluded that the color changes of smart labels were consistent with three different stages of freshness, including fresh step (reddish purple), medium step (brown), and spoiled step (yellow), which could be recognized by naked eyes. Eskandarabadi et al. [66] designed the intelligent ethylene-vinyl acetate nanocomposite film with different additives such as rosemary extract, anthocyanin extract, and ZnO/Fe-MMT nanoparticles to detect meat deterioration. They investigated the color change behavior of anthocyanin at different pH levels. The meat deterioration was detected due to the released ammonia, causing the color change of films from red (acidic color) to yellow (basic color). Wu et al. [52] prepared and tested a smart system using the incorporation of oxidized chitin nanocrystals and RCA into konjac glucomannan films (KCR). Based on the obtained results, with an increase in pH value, the KCR film’s color turned from pink–red to green. The intensity of this color change was significantly dependent on the incorporation amount of anthocyanin (Figure 3). 

### 4.3. Extraction Factors Affecting the Intelligent Characteristics of Bio-Based Films

Anthocyanins extracted from different sources can indicate various colors and stability modes because of the intrinsic characteristics in their chemical configurations. However, studies on the effect of temperature, light, and time on the stability of pH-sensitive indicators are currently limited. Meanwhile, the functional properties (antioxidant/antimicrobial activity and pH sensitivity) of anthocyanins-rich films mainly depend on the stability and release of anthocyanins from the composite film matrix, which is attributed to their concentration, microstructure of films, and intermolecular interactions of anthocyanins with the film matrix [76,77]. In one of the studies, Prietto et al. [54] prepared pH-sensitive packaging films based on corn starch comprising RCAs and black bean anthocyanins (BBAs). They observed that BBAs are more sensitive against different pHs and exhibit color changes very rapidly so that the appearance changed within about 5 s. In contrast, the pH-sensitive films with RCAs presented higher stability and greater color variation compared with BBAs. They concluded that RCAs are suitable candidates to fabricate intelligent packaging films. In a study conducted by Hamzah et al. (2021), the color change of sago starch-based film (with different concentrations) indicated that the RCA was released from the film into the water during 32 h with different colors on release, which was observed as an increasing trend in color intensity. The authors also claimed the color change was observed among pHs 4, 9, and 13. The release of color from the films shows the stability of anthocyanin which plays a key role in the efficiency of smart packaging films. Thus, if the anthocyanins are not appropriately sustainable/compatible with the film structure, the packaging system will face some challenges to be regarded as an intelligent packaging system [67].

The extraction procedure of RCA is also an important factor in pH sensing. Musso et al. [64] prepared gelatin-based smart films using RCA and claimed that the anthocyanin-rich film was pink at pH < 4 but turned yellow at pH > 11. They reported that the alcoholic extract had a purple coloration while the aqueous one had a pink hue. In addition, the alcoholic-extracted anthocyanins present better functionalities and higher effectiveness as a result of higher contents and varieties of anthocyanins as compared with aqueous-extracted anthocyanins. Thus, the gelatin/anthocyanin film showed pH sensitivity and strong antioxidant activity that were associated with the extraction conditions of anthocyanins. 

### 4.4. Intelligent Characteristics in Electrospun Fibers

In recent years, electrospun nanofibers prepared through the electrospinning technique have been utilized in active and intelligent food packaging systems equipped with halochromic indicators. In a study, Maftoonazad and Ramaswamy [58] produced an electrospun nanofiber mat based upon PVA, and RCE as a pH-biosensor to monitor the pH-dependent quality attributes of rutab (a kind of soft date fruit). Based on the obtained results, relying on the temperature fluctuations over the storage, the pH variations could be correlated to the physiological activity of the fruit itself, and metabolites generated during the microbial growth. At 25 °C, the pH of rutab strongly decreased during 96 h of storage (complete fruit spoilage), causing the mat color to alter slightly after 72 h and was completely changed to purple after 96 h. However, at 5 °C, the pH value gradually dropped, and the color completely changed to purple after 12–20 days. As a result, the pH biosensor can be used as a real-time pH indicator to monitor the progression of spoilage of packaged rutab. Safitri et al. [69] also incorporated the RCA into the poly (ethylene glycol) diacrylate-based hydrogel containing lignocellulose nanofiber (PEGDA/LCNF) hydrogel as a colorimetric pH indicator film, which successfully indicated multicolor response at specific pH buffers. Pourjavaher et al. [57] prepared a pH indicator based on BC nanofibers by adding RCA. The BC-diluted anthocyanin label exhibited the highest response to the tested range of pH (pH 2–10). However, labels containing concentrated anthocyanins indicated to be the least sensitive to the pH variations. Prietto et al. [71] developed halochromic electrospun fibers based on zein (30% *w*/*v*) and RCAs (3% *w*/*v*, 4% *w*/*v*, and 5% *w*/*v*), which showed a color alteration in fibers from red to green by enhancing pH from 1 to 10. The results also displayed that an increase in the concentration of RCAs from 3 to 5% led to an increase in the intensity of color variation at different pH values. 

### 4.5. pH-Sensing Applications in Health Monitoring

In addition to food packaging, the RCA-rich electrospun biopolymers could act as a colorimetric pH sensor in the health monitor. Pakolpakçıl et al. [72] also showed that the pH-responsive electrospun nanofibers based on the PVA (12% *w*/*w*), sodium alginate (1% *w*/*w*), and red cabbage extract (2–3%) display color differences from red to pink, blue, and finally to green by increasing pH from 4 to 10, which potentially can be used for monitoring wound healing. In another study, Devarayan and Kim fabricated an eco-friendly and reversible pH sensor by immobilizing RCAs on electrospun cellulose acetate fiber mats as a potential substitute for diagnosing the alcoholic individuals and monitoring the evolution of certain illnesses. The effectiveness of pH-responsive nanofibers was evaluated after exposure to different temperatures and pH values during storage for 30 days. No significant differences were found in color responses of composites after 1 day of storage at −50 °C compared with the fresh samples. While the color response pattern was different after treatment at 100 °C. After one month, the responsiveness of the pH sensor did not change. Thus, it was confirmed that the pH sensor was constant at different temperatures and storage times, with the capability of sensing pH in the range of 1–14. Additionally, the sensors were reversible when dipped in different buffer solutions [70]. 

### 4.6. Active Characteristics in Bio-Based Films

In addition to the pH-responsive color-changing properties of RCAs for monitoring the freshness or spoilage of food products in the intelligent packaging, they could provide new opportunities for the development of active packaging for extending the shelf life of the packaged products. Some studies described the acceptable antioxidant and antimicrobial activities of RCAs-rich films [50,52,53,64,66]. For instance, Chu et al. [63] indicated that the diameter of the inhibition zone of RC3 film (CGG-HEC10 with 3% RCA) against *E. coli* and *S. aureus* was higher than that of the CGG–HEC10 composite film (without RC). Thus, RC3 film represented good antibacterial activity because of the presence of red cabbage pigments. Similar results were obtained by Wu et al. [52], who observed an RCA concentration-dependent antimicrobial activity in KCR films against *E. coli* and *S. aureus*. They also observed that the DPPH radical scavenging ability increased with increasing RCA concentration in KCR films due to the strong antioxidant activity of anthocyanins. Furthermore, Eskandarabadi et al. [66] detected the highest antioxidant activity against DPPH in ethylene-vinyl acetate films with RCA stabilized on montmorillonite compared with the other active materials such as ZnO nanoparticles and rosemary extract. In addition, RCA films had higher antimicrobial activities against *E. coli* and *S. aureus* than the ZnO film because of the higher penetration rate of anthocyanins [66].

The extraction procedure of RCAs from red cabbage also affects the active capabilities of packaging. Musso et al. reported the significantly increased antioxidant properties (by ABTS^.+^ and FRAP assays) of the gelatin films incorporating the aqueous (GAw) and alcoholic (GAe) RCE. In addition, the antioxidant activity of GAe was remarkably higher than GAw. This difference could be related to the higher content and varieties of anthocyanins in alcoholic red cabbage extract. However, in this work, the addition of RCE (extracted with different solvents) showed no significant antimicrobial activity against different bacterial strains [64]. This could probably be attributed to the low concentration of anthocyanins in both extracts and also specific interactions between antimicrobial agents of anthocyanins and gelatin in the film matrix.

## 5. Concluding Remarks

Smart food packaging is all about ingenious procedures to protect food items by choosing rapid, cost-effective, and efficient techniques to monitor food quality over shelf life and transportation. The key benefits of smart packaging systems and sensors are suitable traceability via meta information and serialized packages, facilitated quality and safety management by providing real-time data about the food products, reduced lab and analysis costs, linking food processors to the market and customers, etc. Recently, the incorporation of red cabbage anthocyanins (RCAs) into biopolymer-based films has provided new prospects for active and intelligent food packaging applications. Loading RCA into the matrix of packaging films often causes different changes in their physical, mechanical, thermal, and structural properties, mainly due to the formation of inter- and intra-molecular connections (e.g., electrostatic interactions and hydrogen bonds) between polymer functional groups and hydroxyl groups in anthocyanins. As a result, smart biopolymeric films containing RCAs have a bright future in the packaging industry to prolong the shelf life of food products, monitor the food freshness and quality, and improve product and customer safety. Smart packaging systems could also be used as commanding tools for addressing food safety management systems. However, the commercialization of smart food packaging systems faces several challenges in terms of regulations (labeling), cost, sustainability, security and data privacy, and acceptance by the supply chain and customers as end-users. 

## Figures and Tables

**Table 1:** Halochromic composite films based on red cabbage anthocyanin: physical and mechanical changes.

Biopolymer/ Polymer	Application	pH Values/Color Change	Main Results after RCAs Incorporation			References
			Physical Properties	Mechanical Properties		
				TS	EAB	
Bacterial cellulose membrane	Intelligent packaging film for milk	pH = 1–14, color variation from red to purple, gray, and then to yellow 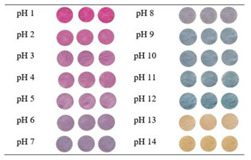	Significant increase in water solubility and swelling index	↓	↑	[19]
Sago starch	Intelligent packaging film	pH = 1–13, clearly color changes between pH 4, 9 and 13 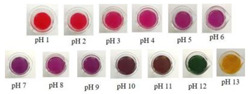	Decrease in moisture content	_	_	[67]
Ethylene-vinyl acetate/ZnO/Fe-MMT nanoparticles	Smart packaging film	pH = 2–12, red to yellow 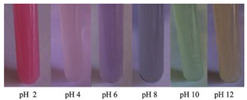 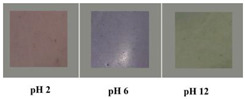	_	_	_	[66]
Green banana starch/gelatin/alginate	Intelligent packaging film for sheep meat	pH = 2–13 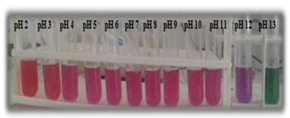	Decrease in thickness, swelling Index and WVP	_	_	[56]
ASK Gum/CMC·Na	Intelligent packaging film	pH = 3–10 (pink to green) (pH = 3 (rose–bengal), pH = 4–6 (purple), pH = 7 (bluish black), pH = 8–9 (atropurpureus), and pH = 11 (aquamarine)) 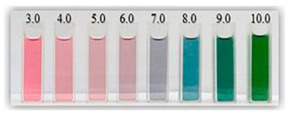	Increase in WVP and light transmission, transparency; increase in OP value with increases in RCA content from 5 to 15%	↓	↑	[41]
Corn starch	Intelligent packaging film	pH = 1–10, color variation from pink to purple and blue depending on the pH variations 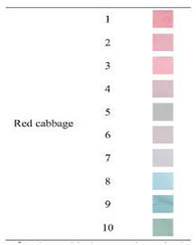	Increase in thickness and water solubility	↔	↔	[54]
Bovine gelatin	Smart packaging film	pH = 1–14	No effect on thickness and WVP; decease in moisture content; increase in water solubility (alcoholic extract)	Alcoholic extract: (↔); Aqueous extract: (↑)	Alcoholic extract: (↑); Aqueous extract: (↔)	[64]
Bovine gelatin	Smart packaging film	pH = 2–12, purple, blue, and finally green at pH 8–12 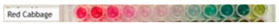	No effect on the thickness	_	_	[50]
KGM/O-ChNCs	Smart packaging film	pH = 2–12, the color changed from pink–red to green 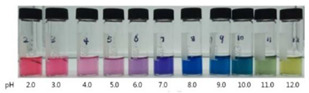	No significant effect on thickness; increase in water solubility and WVP; Decease in light transmittance	↓	↓	[52]
Hydroxypropyl methylcellulose	Intelligent packaging film	pH = 2–9 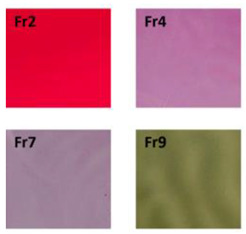	No effect on thickness; changes in the light barrier properties	↓	↑	[51]
CBO-loaded chitosan capsules	Smart packaging film for fish	pH range of 6–8 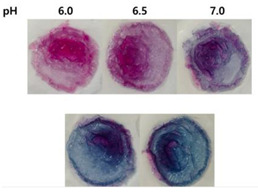	No effect on the thickness	↓	↑	[53]
Bacterial cellulose nanofibers	Intelligent packaging film	pH = 2–10, color change in the BCA label at pH 2 and 3 (dark red) and pH 4–10 (dark violet), and in the BCDA label from bright red to dark blue 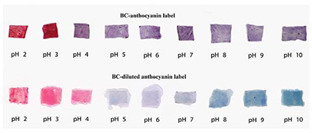	Increase in moisture absorption	↓	↑	[57]
Dual-modified cassava starch	Intelligent packaging film	pH = 2–12 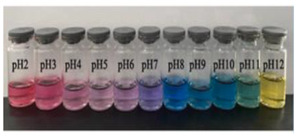	_	_	_	[68]
Chitosan/oxidized–chitin nanocrystals	Smart packaging film for hairtail and shrimp	pH = 3–10, color variations (red–pink–purple–blue–green) in different pHs 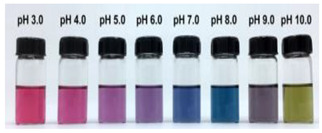	No effect on thickness; significant decrease in WVP, OP values, and light transmittance	↓	↑	[39]
Chitosan/PVA	Intelligent packaging film for pork meat	pH = 1–13 (pH = 1 (reddish color), pH ≈ 6 (purple), pH = 7–8 (blue), pH = 9 (sea green), pH ≈ 12 (yellow–green)) 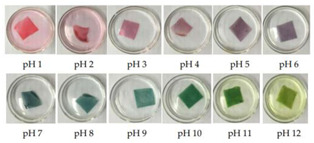	_	↑	↑	[65]
Cationic guar gum film/hydroxyethyl cellulose	Smart packaging film for pork meat and soybean milk (SBM)	Pork for 72 h and SBM for 18 h 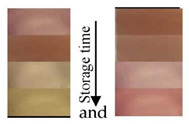	Decrease in WVP and OP; decrease in light transmittance	(↑) with ≤3% RCA, (↓) with 5% RCA	(↑) with ≤3% RCA, (↓) with 5% RCA	[63]
Chitosan/corn starch	Intelligent packaging film for fish fillet	pH = 2–13 Blue after 16 h, and yellow after 72 h at room temperature	No significant change in WVP	_	_	[45]
PVA	Intelligent electrospun nanofiber mat for packaging date fruit (rutab)	pH = 2–12 At 25 °C: after 72 h (color altered slightly), 72–96 h (violet and purple), after 96 h (purple) At 5 °C: color completely changed to purple after 12–20 days 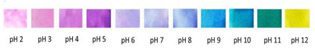	Increase in moisture adsorption	↓	↑	[58]
PEGDA/LCNF	Intelligent hydrogel	pH = 1–14 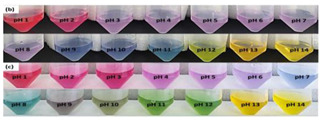 pH = 7–14 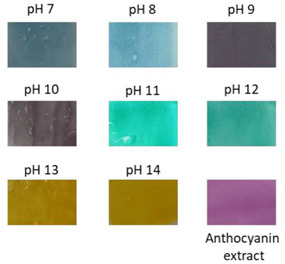	No significant change in moisture adsorption	_	_	[69]
Cellulose acetate	Intelligent electrospun Nanofiber for health monitor	pH = 1–14 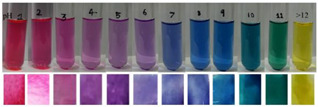	_	_	_	[70]
Zein	Intelligent electrospun fiber	pH = 1–14 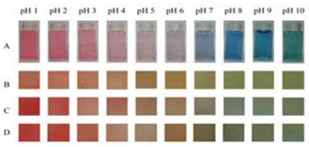	_	_	_	[71]
PVA/NaAlg	Intelligent electrospun nanofiber for wound dressing	pH = 4–10 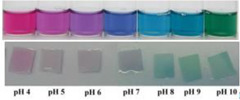	_	_	_	[72]
PVA/sodium carboxymethyl cellulose	Intelligent packaging film for pork meat	Pork for 24 h 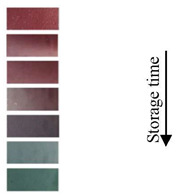	Increase in thickness	↓	↑	[55]
Cellulose acetate	Intelligent packaging film	pH = 1–12 (red color at pH 1–2, pink at pH 3, violet at pH 4–6, blue at pH 7–8, green at pH 9–11, and yellow at pH 12) 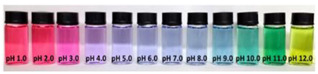	Increase in thickness and OP; decrease in light transmittance	↓	↑	[47]

CBO: clove bud oil, PVA: polyvinyl alcohol, SBM: soybean milk, KGM: konjac glucomannan, ASKG: Artemisia sphaerocephala Krasch. gum, O-ChNCs: oxidized chitin nanocrystals, CMC·Na: carboxymethyl cellulose sodium, BCA: BC–anthocyanin, BCDA: BC-diluted anthocyanin, PEGDA: poly (ethylene glycol) diacrylate, LCNF: lignocellulose nanofiber, NaAlg: sodium alginate, NA: not affected, WVP: water vapor permeability, and OP: oxygen permeability. The arrows ↑, ↓ and ↔ indicate significant increase and decrease and no significant change, respectively. All figures/tables used in this table are reprinted with permission from their publishers where needed.

## Data Availability

Not applicable.
